# Comparative Pharmacodynamics of Three Different Botulinum Toxin Type A Preparations following Repeated Intramuscular Administration in Mice

**DOI:** 10.3390/toxins14060365

**Published:** 2022-05-25

**Authors:** Jaeyoon Byun, Seongsung Kwak, Jin-Hee Kwon, Minhee Shin, Dong-Kyu Lee, Chang-Hoon Rhee, Won-ho Kang, Jae-Wook Oh, Deu John M. Cruz

**Affiliations:** 1 Medytox Gwanggyo R&D Center, 114 Central town-ro, Yeongtong-gu, Suwon-si 16506, Korea; bjy2689@medytox.com (J.B.); sskwak@medytox.com (S.K.); jinhee5761@medytox.com (J.-H.K.); minishin@medytox.com (M.S.); dongkyulee@medytox.com (D.-K.L.); whkang@medytox.com (W.-h.K.); 2 Department of Stem Cell and Regenerative Biotechnology, Konkuk University, 120 Neungdong-ro, Gwangjin-gu, Seoul 05029, Korea; 3 Medytox Osong R&D Center, 102 Osongsaengmyeong 4-ro, Osong-eup, Heungdeok-gu, Cheongju-si 28161, Korea; chrhee@medytox.com

**Keywords:** botulinum toxin, digit abduction score, compound muscle action potential, repeated injection, duration of action

## Abstract

Botulinum neurotoxin type A (BoNT/A) causes muscle paralysis by blocking cholinergic signaling at neuromuscular junctions and is widely used to temporarily correct spasticity-related disorders and deformities. The paralytic effects of BoNT/A are time-limited and require repeated injections at regular intervals to achieve long-term therapeutic benefits. Differences in the level and duration of effectivity among various BoNT/A products can be attributed to their unique manufacturing processes, formulation, and noninterchangeable potency units. Herein, we compared the pharmacodynamics of three BoNT/A formulations, i.e., Botox^®^ (onabotulinumtoxinA), Xeomin^®^ (incobotulinumtoxinA), and Coretox^®^, following repeated intramuscular (IM) injections in mice. Three IM injections of BoNT/A formulations (12 U/kg per dose), 12-weeks apart, were administered at the right gastrocnemius. Local paresis and chemodenervation efficacy were evaluated over 36 weeks using the digit abduction score (DAS) and compound muscle action potential (CMAP), respectively. One week after administration, all three BoNT/A formulations induced peak DAS and maximal reduction of CMAP amplitudes. Among the three BoNT/A formulations, only Coretox^®^ afforded a significant increase in paretic effects and chemodenervation with a prolonged duration of action after repeated injections. These findings suggest that Coretox^®^ may offer a better overall therapeutic performance in clinical settings.

## 1. Introduction

Botulinum neurotoxins (BoNT) are highly potent exotoxins produced by *Clostridium botulinum*, as well as a few related species, and are well-known to cause muscle paralysis by blocking the release of acetylcholine from presynaptic cholinergic nerve endings at neuromuscular junctions (NMJ) [1]. The active form of all BoNT consists of two polypeptides, a 100 kDa heavy chain (Hc) and a 50 kDa light chain (Lc), linked via a disulfide bond. The Hc facilitates BoNT binding to the synaptic vesicle protein 2 (SV2) and polysialogangliosides at the presynaptic membrane of nerve terminals, after which the holotoxin gets internalized by vesicle endocytosis [2,3]. Protonation of the vesicle unfolds the holotoxin, enabling the Hc to act as both protein-conducting channel and transmembrane chaperone that allows membrane translocation of the Lc into the cytoplasm [4]. During translocation across the endosomal membrane, the interchain disulfide bond gets reduced by the theoredoxin-theoredoxin reductase system (Trx-TrxR) facilitating the separation of the Lc from the Hc, while the chaperon protein Hsp90 mediates refolding of the Lc to its native fold [5,6,7]. After reacquiring its active state, the Lc is now able to cleave components of the SNARE protein complex (SNAP-25, syntaxin, synaptobrevin) which subsequently interrupts acetylcholine transport from the cytosol into the synaptic cleft [8,9,10].

BoNT is traditionally classified into seven antigenically distinct serotypes (A, B, C, D, E, F, and G), and different subtypes in some of these serotypes [11,12]. In recent years, new serotypes and chimeras of existing BoNTs have been identified, such as BoNT/X, BoNT/H (hybrid of BoNT/A1 and BoNT/F5), and BoNT/CD (mosaic of BoNT/C and BoNT/D) [13,14,15,16]. In addition, the discovery of “botulinum-like” toxins in non-clostridial species have intrigued scientists on the evolutionary origin of this highly potent neurotoxin [17,18]. In 1980, BoNT type A (BoNT/A) became the first BoNT approved for human use as an alternative to surgical manipulation to treat strabismus in pediatric patients [19]. Since then, clinical indications for the use of BoNT/A have expanded to a wide variety of focal hyperkinetic movement disorders, focal and segmental upper- and lower-limb spasticity, and other neurological disorders [20,21]. Moreover, BoNT/A is widely employed for facial aesthetic treatment and is a popular anti-wrinkle agent. There are eight known subtypes of BoNT/A (A1~A8), and BoNT/A1 is the most commonly used drug product for pharmaceutical purposes, owing to the extensive studies at molecular and cellular levels, as well as preclinical and clinical investigations [12,22].

BoNT/A is an effective and generally safe treatment for patients who suffer from chronic conditions associated with neuromuscular disorders such as hemiplegic cerebral palsy, cervical dystonia, hemifacial spasm, blepharospasm, among others [23,24]. However, BoNT/A treatment failure and secondary non-responsiveness following repeated administration have been reported [25,26,27,28]. Therefore, repeated BoNT/A administration should maintain the desired treatment effects while avoiding potential side effects and conditions that elicit secondary non-responsiveness [29]. Several studies have investigated the local effects and effective duration of BoNT/A in rodents, although most reported their findings performed only 1 or 2 injections of BoNT/A formulations. Moreover, reports on the effects of neutralizing antibody production following repeated BoNT/A administration are lacking [30,31,32,33,34].

Several different BoNT/A products are available in the market, including Coretox^®^ (Medytox Inc., Cheongju-si, Korea), Xeomin^®^ (Merz Pharmaceuticals GmbH, Frankfurt am Main, Germany), and Botox^®^ (Allergan, Irvine, CA, USA). Botox^®^ contains neurotoxin complexes, whereas Coretox^®^ and Xeomin^®^ contain a complexing protein-free neurotoxin. Although all these formulations contain the 150 kDa core neurotoxin, intrinsic, and extrinsic factors, including their unique manufacturing process, excipients, and potency units, potentially contributing to differences in the clinical effectiveness and duration of BoNT/A formulations, consequently influencing the dosage and dosage interval needed to achieve the desired therapeutic benefits [35,36].

We have previously compared the pharmacodynamics of three different BoNT/A formulations (Botox^®^, Xeomin^®^, and Coretox^®^) in ICR mice using a single intramuscular (IM) injection [32]. In the present study, the pharmacodynamics of these three BoNT/A formulations were compared by administering three repeated IM injections into the right gastrocnemius of ICR mice at 12-week intervals. The 12 U/kg IM dose was used in this study as it has been shown previously that this dosage induces strong paretic and chemodenervation effects without eliciting serious adverse events in the animals [32]. The pharmacological activity and duration of action of each BoNT/A formulation were evaluated over 36 weeks by grading the level of paresis over time and monitoring changes in the motor endplate potential on ipsilateral hind limbs.

## 2. Results

### 2.1. Paretic Effects following Repeated IM Injections of BoNT/A

Paresis of both hind limbs of mice was graded using the digit abduction score (DAS), a physiological model that evaluates muscle flaccidity. A DAS value of 0 indicates full digit abduction, whereas a value of 4 represents the absence of abduction and leg extension, indicating complete muscle paralysis [37]. The right (ipsilateral) hind limb of treated animals exhibited varying degrees of muscle weakening following each repeated IM injection of Coretox^®^, Xeomin^®^, or Botox^®^ at 12 U/kg per dose (potency units based on the product label of each BoNT/A formulation) during the 36-week evaluation period, as determined by the loss of digit abduction. Conversely, the left (contralateral) hind limb of the same animals showed no signs of muscle weakening at any examined time point (data not shown), suggesting that all three BoNT/A formulations only induce local paresis at the given dosage.

#### 2.1.1. Comparison of Local Paresis among the Three BoNT/A Formulations

Local paretic effects resulting from each repeated IM injection of Coretox^®^, Xeomin^®^, and Botox^®^ were compared using the following parameters: peak DAS (DAS_max_), duration of action, and cumulative DAS over time using the area under the curve (DAS_AUC_). The DAS profile of each BoNT/A formulation is summarized in Table 1. The average DAS_max_ values were comparable among the three BoNT/A formulations following the first dosing cycle, ranging between 2.8 and 3.1. Following the second dosing cycle, Xeomin^®^ induced a lower average DAS_max_ value than that of Coretox^®^. Likewise, Xeomin^®^ showed a significantly lower average DAS_max_ value than that of Botox^®^ and Coretox^®^ following the third dosing cycle. Considering the effective duration and cumulative DAS over time, the three BoNT/A formulations showed no significant difference in duration of action during the first dosing cycle (35.0~45.4 days). However, Xeomin^®^ exhibited a significantly lower DAS_AUC_ than that of Coretox^®^. During the second dosing cycle, Coretox^®^ demonstrated a significantly longer duration of action and larger DAS_AUC_ than that induced by Xeomin^®^. Following the third dosing cycle, Coretox^®^ elicited a significantly longer duration of action and larger DAS_AUC_ than that of both Xeomin^®^ and Botox^®^.

#### 2.1.2. Course of Paretic Effects following Repeated IM Injections of Each BoNT/A

**Figure 1:**
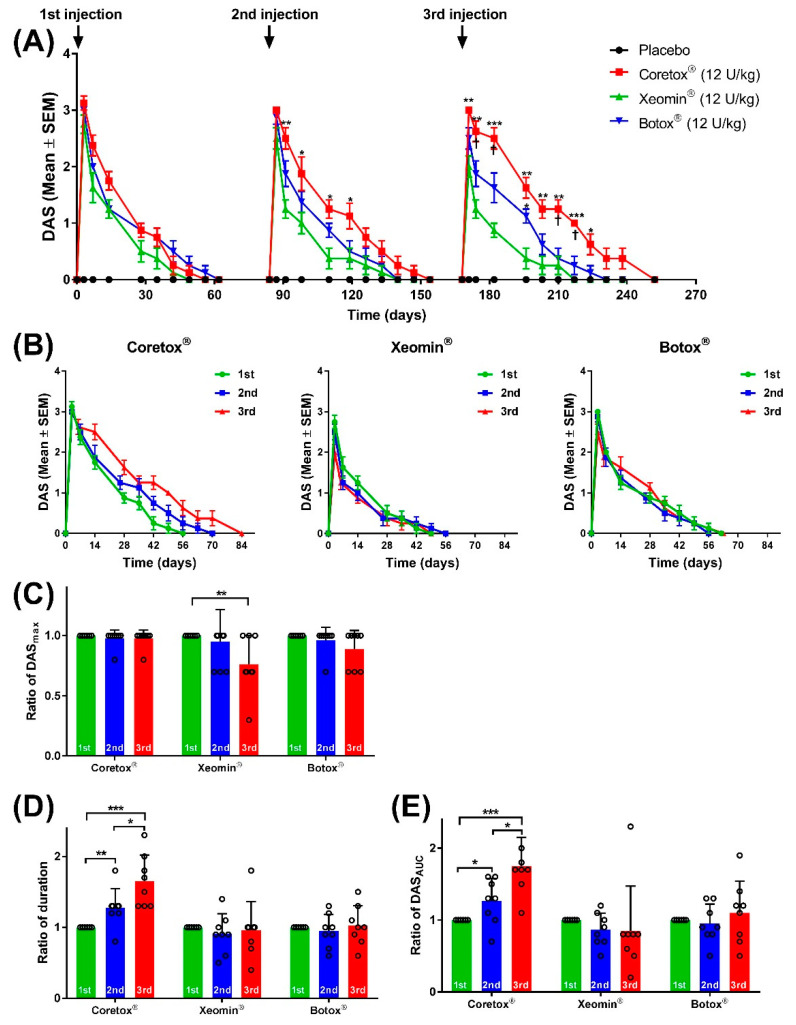
(**A**) Time course of local paresis on the ipsilateral hind limb of mice based on digit abduction score (DAS) following repeated intramuscular (IM) injections of BoNT/A formulations administered to the right gastrocnemius. (**B**–**E**) Graphs showing changes in DAS values (**B**); ratio of DAS_max_ (**C**), duration of action (**D**), and DAS_AUC_ (**E**) of the BoNT/A formulations after each repeated IM injection (12 weeks interval) relative to the first injection. Paretic effects are graded using a modified DAS criterion (0 to 4, increasing magnitude). (**A**,**B**) Data points represent mean DAS ± SEM at different time points (n = 8 per group on the first dosing cycle; n = 7~8 per group on the second dosing cycle; n = 6~8 per group on the third dosing cycle). (**C**–**E**) Dot plot represents individual values with columns and error bars representing mean ± standard deviation. (**A**) * *p* < 0.05, ** *p* < 0.01, *** *p* < 0.001 between Xeomin^®^ and Coreotox^®^ or Xeomin^®^ and Botox^®^, and ^†^ *p* < 0.05 between Coretox^®^ and Botox^®^ by Mann–Whitney test. (**C**–**E**) * *p* < 0.05, ** *p* < 0.01, *** *p* < 0.001 between repeated doses by Student’s *t*-test. BoNT/A, botulinum neurotoxin type A.

Figure 1A presents the course of paretic effects on the ipsilateral hind limb following three repeated IM injections of Botox^®^, Xeomin^®^, and Coretox^®^ (12 U/kg per dose). Considering all three BoNT/A formulations, peak DAS values were observed three days after each repeated injection, followed by a steady decline over time. At the start of the next dosing cycle, all animals had reached full recovery (DAS = 0). Compared with the other two BoNT/A formulations, the duration of paretic effects of Coretox^®^ increased after successive repeated doses (Figure 1A,B). Changes in the level of local paretic effects induced by the first, second, and third IM injections with each BoNT/A formulation were evaluated by comparing the average ratio of DAS_max_, DAS_AUC_, and duration of action of the second and third dosing cycles to the first dosing cycle. Botox^®^ and Coretox^®^ showed no significant difference in the ratio of DAS_max_ between the three repeated injections, whereas Xeomin^®^ presented a significantly reduced ratio of DAS_max_ after the third injection (Figure 1C). Both Botox^®^ and Xeomin^®^ showed no significant changes in the duration of action and DAS_AUC_ in all three dosing cycles. Meanwhile, Coretox^®^ displayed a significant increase in both duration of action and DAS_AUC_ value after each successive injection (Figure 1D,E).

### 2.2. Chemodenervation on the Ipsilateral Hind Limb following Repeated IM Injections of BoNT/A

In addition to assessing paresis using DAS, we evaluated the efficacy of the BoNT/A formulations to induce chemodenervation on the ipsilateral hind limb of mice following IM injection into the right gastrocnemius by performing a compound muscle action potential (CMAP) assay, electromyography measuring summated action potentials of stimulated motor endplates from several muscle fibers within the same area. A reduction in CMAP can be correlated with the biological activity of neuromuscular blocking agents, including BoNTs [31].

#### 2.2.1. Comparison of Chemodenervation among the Three BoNT/A Formulations

Chemodenervation induced by Coretox^®^, Xeomin^®^, and Botox^®^ after each repeated IM injection was compared using the following parameters: CMAP amplitude at the start of the dosing cycle (CMAP_baseline_), lowest CMAP (CMAP_min_), and cumulative CMAP losses over time using the area above the curve (CMAP_AAC_). The CMAP profile of each BoNT/A formulation is summarized in Table 2. Considering the three BoNT/A formulations, Xeomin^®^ induced significantly weaker chemodenervation, as shown by the higher CMAP_min_ in all three dosing cycles. Coretox^®^ and Botox^®^ induced CMAP_min_ values ranging from 1.43~1.45 mV in the first dosing cycle, 1.46~1.61 mV in the second dosing cycle, and 0.58~1.11 mV in the third dosing cycle, whereas Xeomin^®^ induced CMAP_min_ values of only 3.49 mV, 3.05 mV, and 3.19 mV in the first, second, and third dosing cycles, respectively. Additionally, Xeomin^®^ consistently exhibited significantly lower CMAP_AAC_ values than those of Coretox^®^ and Botox^®^ in all three dosing cycles. Conversely, Coretox^®^ showed a significant increase in CMAP_AAC_ when compared with both Botox^®^ and Xeomin^®^ by the third dosing cycle.

#### 2.2.2. Course of Chemodenervation following Repeated IM Injections of Each BoNT/A

**Figure 2:**
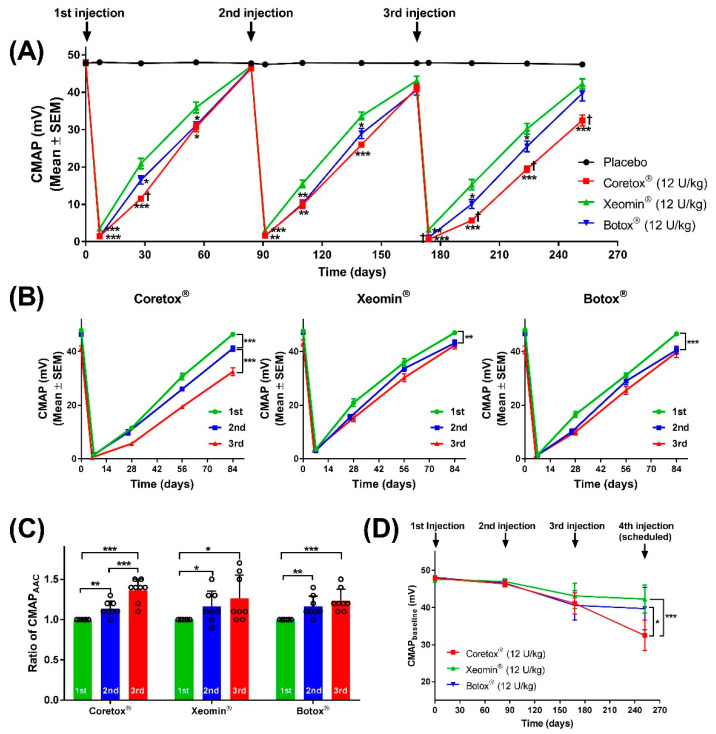
(**A**) Time course showing changes in the compound muscle action potential (CMAP) of the ipsilateral hind limb of mice following repeated intramuscular (IM) injection of BoNT/A formulations administered to the right gastrocnemius. (**B**–**D**) Graphs showing changes in CMAP (**B**); ratio of CMAP_AAC_ (**C**), and CMAP_baseline_ (**D**) after each repeated IM injection (12 weeks interval) relative to the first injection. (**A**,**B**) Data points represent mean CMAP ± SEM in millivolts (mV) at different time points (n = 8 per group on the first dosing cycle, Days 0~84; n = 7~8 per group on the second dosing cycle, Days 84~168; n = 6~8 per group on the third dosing cycle, Days 168~252); error bars not included for clarity. (**C**) Dot plot represents individual values with columns and error bars representing mean ± standard deviation. (**D**) Data points and error bars represent mean ± standard deviation. (**A**) * *p* < 0.05, ** *p* < 0.01, *** *p* < 0.001 between Xeomin^®^ and Coreotox^®^ or Xeomin^®^ and Botox^®^, and ^†^ *p* < 0.05 between Coretox^®^ and Botox^®^ by student’s *t*-test. (**B**–**D**) * *p* < 0.05, ** *p* < 0.01, *** *p* < 0.001 between repeated doses by Student’s *t*-test. BoNT/A, botulinum neurotoxin type A.

Figure 2A presents the time course of changes in the average CMAP of the ipsilateral hind limb following three repeated IM injections of Coretox^®^, Xeomin^®^, and Botox^®^ (12 U/kg per dose). The average CMAP in the placebo control group ranged between 47.48~48.05 mV, and CMAP amplitudes measured from each mouse showed minimal variations (0.61~1.04% coefficient of variation) throughout the study period. In contrast, a significant drop in CMAP amplitudes was observed in Coretox^®^, Xeomin^®^, and Botox^®^ treatment groups one week after each repeated IM injection, followed by a steady recovery. Maximum chemodenervation was observed 6~7 days after each repeated IM injection, with reduced CMAP amplitudes exceeding 90%. Similar to the course of paretic effects, chemodenervation effects induced by each repeated dose of BoNT/A formulations were reduced over time. At the end of the first dosing cycle, the average CMAP reverted to near pre-injection levels. However, the average CMAP at the end of the second and third dosing cycles did not approach pre-injection levels. Based on the CMAP curves, we extrapolated that the recovery period to pre-injection levels was longer in the Coretox^®^ administered group than in Xeomin^®^ and Botox^®^ administered groups at the third dosing cycle.

We also compared chemodenervation resulting from repeated IM injections for each BoNT/A formulation. Unlike Xeomin^®^ and Botox^®^, the estimated duration of action of Coretox^®^ following the third repeated IM injection significantly increased, as determined by extrapolation from CMAP curves (Figure 2B). Relative to their first injection, all three BoNT/A formulations showed a significant increase in the ratio of CMAP_AAC_ following the second and third injections. However, only Coretox^®^ demonstrated a significant increase in the ratio of CMAP_AAC_ between the second and third injections, largely due to the prolonged duration of action from the latter (Figure 2C). Differences in the duration of action between repeated IM injections for each BoNT/A formulation could not be accurately determined, given that the dosing interval of 12 weeks precluded the hind limbs from reaching full recovery (pre-injection CMAP amplitudes). Furthermore, if another repeated injection was administered following the same dosing interval of 84 days, the CMAP_baseline_ for the fourth dosing cycle, represented by the CMAP measurement at Day 252, would show that Coretox^®^ exhibited the most persistent chemodenervation among the three BoNT/A formulations (Figure 2D).

## 3. Discussion

BoNT/A is a highly effective presynaptic neuromuscular blocking agent and has been approved as a first-line treatment for various indications such as strabismus, hemifacial spasm, cervical dystonia, limb spasticity, cerebral palsy, chronic migraine, axillary hyperhidrosis and overactive bladder [20,21,38,39]. However, like most drugs, the pharmacological effect of BoNT/A is temporary, and patients with chronic conditions require repeated injections at specific intervals for continuous therapeutic benefits [26,40]. Several BoNT/A products available in the market have received regulatory approval for repeated administration of therapeutic doses at approximately 10- to 12-weeks intervals. More recently, it has been proposed that a more flexible and patient-tailored dosing regimen needs to be implemented for certain indications based on differences in the potency of each BoNT/A formulation [41].

In the present study, we compared the pharmacodynamics of Coretox^®^, Xeomin^®^, and Botox^®^ on the ipsilateral hind limbs of mice when administered to the right gastrocnemius at regular intervals (12 weeks) using the same dosage (12 U/kg per IM injection) based on the labeled potency. Following the first injection, the onset and duration of local paresis and chemodenervation were comparable among all three BoNT/A formulations. However, Xeomin^®^ exhibited lower cumulative effects for both paresis and chemodenervation than those of Coretox^®^, similar to previously reported findings [32]. By the third repeated injection, Coretox^®^ showed prolonged paretic effects coinciding with increased cumulative chemodenervation. Both Xeomin^®^ and Botox^®^ elicited a comparable duration of paretic effects from the first to the third injections, despite the enhanced cumulative reduction in motor endplate potential by the latter dosing cycle. Comparing DAS and CMAP profiles, Coretox^®^ showed superior performance over Xeomin^®^ and Botox^®^ in paretic effects and chemodenervation following repeated IM injections in mice assessed under identical conditions.

The gradual increase in CMAP_AAC_ observed in mice following repeated administration of the BoNT/A formulations corroborated with a previous report describing the events at the NMJ of muscle fibers from mouse epitrochleoanconeus following repeated administration of BoNT/A. The authors showed that repeated BoNT/A exposure resulted in longer recovery periods following subsequent injections and attributed this phenomenon to structural abnormalities in individual NMJs, as well as changes in NMJ distribution [34]. Similarly, a clinical study has examined BoNT/A naïve adult patients with post-stroke hemispasticity and spastic foot drop receiving repeated BoNT/A injections every three months for one year. The authors reported a significant increase in treatment benefits after repeated injections when compared with the initial treatment [42].

Conversely, secondary non-responsiveness has been reported in some patients following repeated BoNT/A injections, which may involve the production of neutralizing antibodies against the neurotoxins [43,44]. Repeated BoNT/A injections are critically important for patients with intractable muscle-related conditions. Secondary non-responsiveness can induce serious debilitating effects in patients dependent on repeated BoNT/A treatment to function normally in daily life [45]. However, in the present study, we found no conclusive evidence indicating secondary non-responsiveness attributed to anti-drug antibodies following repeated injections with all three BoNT/A formulations, as determined by a cell-based neutralization assay performed using mouse serum samples (data not shown).

The present study is the first nonclinical report comparing the pharmacodynamics of three BoNT/A formulations (Coretox^®^, Xeomin^®^, and Botox^®^) in ICR mice following three repeated IM injections at 12-week intervals. Coretox^®^, a complexing protein-free neurotoxin, was the only BoNT/A formulation that showed significantly higher efficacy and longer duration of action after repeated injections than that of Xeomin^®^ and Botox^®^. Interestingly, Coretox^®^ is the only BoNT/A formulation that lacks animal-derived components, as it uses polysorbate 20 and L-methionine as excipients, whereas Xeomin^®^ and Botox^®^ formulations contain human serum albumin (Table 3). Further studies are warranted to explore the correlation between formulations and the pharmacodynamics of the BoNT/A products. Furthermore, the present study only investigated the pharmacological effects using a single dosage unit (12 U/kg) and fixed dosing interval (12 weeks); hence, other dosage units and various dosing intervals need to be examined to obtain a more comprehensive comparison of therapeutic effectiveness.

## 4. Conclusions

All examined BoNT/A formulations presented identical pharmacological effects. However, accumulated evidence suggests that differences in formulations may impact therapeutic effectiveness and effective duration of the repeated dosing regimen. Coretox^®^, a complexing-protein-free BoNT/A formulation devoid of animal-derived components, demonstrates significantly higher efficacy and longer duration of action after repeated intramuscular injections compared to Xeomin^®^ and Botox^®^ administered under similar dosing regimen. Expanding our understanding of the physiological events that occur during repeated BoNT/A injections might help provide better guidance for formulating a more therapeutically effective dosing regimen.

## 5. Materials and Methods

### 5.1. Preparation of Botulinum Toxin Type A Formulations

Three different commercial BoNT/A products were procured. OnabotulinumtoxinA (Botox^®^, Allergan, Irvine, CA, USA) is formulated as a 900 kDa BoNT/A complex. The other two products are complexing protein-free 150 kDa BoNT/A, i.e., incobotulinumtoxinA (Xeomin^®^, Merz Pharmaceuticals GmbH, Frankfurt am Main, Germany) and Coretox^®^ (Medytox Inc., Cheongju-si, Korea). A detailed description of each BoNT/A formation is listed in Table 3.

Each BoNT/A formulation was reconstituted to 60 U/mL solutions using preservative-free normal saline (DaiHan Pharm, Ansan-si, Korea). Unopened vials of each BoNT/A formulation were used for each scheduled administration.

### 5.2. Repeated Intramuscular Injection of BoNT/A Formulations

All procedures involving laboratory animals were conducted at the animal facility of Medytox (Gwanggyo, Suwon, Korea) in accordance with the guidelines provided by the Animal Care and Use Committee of Medytox, Inc. (IACUC No. A-2020-016) and in compliance with the Laboratory Animal Act of Korea (Act No. 15278). Six weeks old female ICR/CD-1 mice of approximately 21–30 g (Orient Bio, Gyeonggi-do, Korea) were used in this study. Groups of 8 mice were assigned to each test group designated to receive one of three BoNT/A formulations or normal saline (placebo). Prior to administration, each mouse was anesthetized with an intraperitoneal (IP) injection of 60 mg/kg ketamine hydrochloride (Yuhan Co, Cheongju-si, Korea) and 12 mg/kg xylazine (Bayer, Ansan-si, Korea), and the right hindlimb was shaved. At the start of injection (Day 0), a single IM injection of the BoNT/A formulations was administered to the right gastrocnemius muscle of the corresponding animals at a dose equivalent to 12 U/kg body weight using a 25-μL Hamilton syringe. The placebo control group received a single IM injection of normal saline to the right gastrocnemius at a dose of 0.2 mL/kg body weight. The same procedure was repeated for all groups on Days 84 and 168, performing three repeated injections at 12-week intervals. The animals were housed in a highly controlled environment (23 ± 3 °C, 55 ± 15% relative humidity, 12 h light/dark cycle) with free access to food and water until the end of the study (Day 252). The body weight was measured at various time points, and any animal that showed more than a 20% decrease from their initial body weight at treatment initiation, or manifested signs of moribundity, was immediately euthanized for humane reasons and censored for the rest of the study.

### 5.3. Digit Abduction Score (DAS)

Local paresis of the hind limbs was graded using the modified DAS method as described previously [37]. Briefly, the mouse was suspended by the tail to elicit leg extension and abduction of hind digits. The level of digit abduction on the hind paws was scored by two blinded observers using a 5-point scale from 0 (full digit abduction) to 4 (complete absence of digit abduction and leg extension), with the average of the two observations representing the final score. DAS was recorded prior to and three days after each IM administration of test formulations, as well as at various time points until the end of the study period (Day 252).

### 5.4. Compound Muscle Action Potential (CMAP) Assay

Changes in the motor nerve conduction on the ipsilateral (injected) hind limbs were examined by measuring the CMAP using the Nicolet^®^ VikingQuest™ EMG/NCS/EP system (Viasys Healthcare, Madison, WI, USA) following a previously described method, with some modifications [31]. First, the mouse was anesthetized with a single IP injection of ketamine/xylazine combination as described above and fixed in the prone position after shaving the hind limb. The stimulating electrode (anode) was placed on the right psoas major while the other stimulating electrode (cathode) was placed on the left psoas major, with a grounding electrode placed on the belly of the right rectus femoris. Recording and reference electrodes were placed on the belly of the gastrocnemius and gastrocnemius tendon of the right leg, respectively. Electrical stimulation of approximately 10–20 mA was administered for 0.1 ms, and CMAP amplitude was recorded in millivolts using a filter range of the amplifier fixed to 2–10 K at 60 Hz. CMAP measurement was performed on the hind limb ipsilateral to the injected gastrocnemius before and after 6~7 days of each IM injection, administered either the placebo or BoNT/A formulation, and at various time points until the end of the study period (Day 252).

### 5.5. Statistical Analysis

The response over time curves for DAS and CMAP, as well as summaries for maximum paretic effects (DAS_max_), muscle chemodenervation (CMAP_min_), and duration of action were generated using descriptive statistics. The efficacy of local paresis and chemodenervation induction, represented by DAS_AUC_ and CMAP_AAC_, respectively, was calculated using GraphPad Prism software (version 7.05, GraphPad Software Inc., San Diego, CA, USA). Test for normality by Kolmogorov–Smirnov and Shapiro–Wilk methods was performed using SPSS^®^ statistical software package (version 25.0, SPSS Inc., IL, USA). Comparative analyses between injection cycles of each BoNT/A formulation and between different BoNT/A formulations were examined by one-way ANOVA followed by Tukey’s post hoc test or Mann–Whitney U test using SPSS, or with two-tailed Student’s *t*-test using Microsoft Excel.

## Figures and Tables

**Table 1:** DAS profile of the ipsilateral hind limb of mice after each repeated IM injection of the BoNT/A formulations (12 U/kg per dose) to the right gastrocnemius.

Dosing Cycle	Parameter	Coretox^®^	Xeomin^®^	Botox^®^
First IM Injection	DAS_max_	3.1 ± 0.4 ^a^	2.8 ± 0.5 ^a^	3.0 ± 0.0 ^a^
(Day 0)	Duration (days)	42.0 ± 8.4 ^a^	35.0 ± 8.4 ^a^	45.4 ± 11.0 ^a^
	DAS_AUC_ (DAS·day)	59.4 ± 11.5 ^b^	40.4 ± 16.4 ^a^	55.1 ± 13.8 ^a,b^
Second IM Injection	DAS_max_	3.0 ± 0.0 ^b^	2.5 ± 0.5 ^a^	2.9 ± 0.4 ^a,b^
(Day 84)	Duration (days)	52.5 ± 11.2 ^b^	33.1 ± 14.3 ^a^	41.8 ± 11.0 ^a,b^
	DAS_AUC_ (DAS·day)	75.6 ± 27.6 ^b^	34.7 ± 17.2 ^a^	51.0 ± 17.1 ^a,b^
Third IM Injection	DAS_max_	3.0 ± 0.0 ^b^	2.0 ± 0.5 ^a^	2.6 ± 0.5 ^b^
(Day 168)	Duration (days)	68.3 ± 13.4 ^b^	32.4 ± 11.8 ^a^	44.6 ± 10.5 ^a^
	DAS_AUC_ (DAS·day)	103.4 ± 27.4 ^b^	29.9 ± 16.0 ^a^	57.1 ± 23.2 ^a^

DAS_max_, maximum paresis; duration, duration of action; DAS_AUC_, cumulative DAS per injection cycle; Data represent mean ± standard deviation. One-way ANOVA with Tukey’s HSD test was used (mean values with different superscript letter are significantly different at α = 0.05).

**Table 2:** CMAP profile of the ipsilateral hind limb of mice after each repeated IM injection of the BoNT/A formulations (12 U/kg per dose) to the right gastrocnemius.

Dosing Cycle	Parameter	Coretox^®^	Xeomin^®^	Botox^®^
First IM Injection	CMAP_baseline_ (mV)	47.92 ± 0.59 ^a^	47.66 ± 0.83 ^a^	48.05 ± 0.53 ^a^
(Day 0)	CMAP_min_ (mV)	1.45 ± 0.29 ^a^	3.49 ± 1.21 ^b^	1.43 ± 0.35 ^a^
	CMAP_AAC_ (mV·day)	2045 ± 169 ^b^	1611 ± 264 ^a^	1918 ± 109 ^b^
Second IM Injection	CMAP_baseline_ (mV)	46.40 ± 0.84 ^a^	47.00 ± 0.74 ^a^	46.60 ± 0.92 ^a^
(Day 84)	CMAP_min_ (mV)	1.61 ± 0.86 ^a^	3.05 ± 0.65 ^b^	1.46 ± 0.43 ^a^
	CMAP_AAC_ (mV·day)	2271 ± 156 ^b^	1839 ± 216 ^a^	2198 ± 214 ^b^
Third IM Injection	CMAP_baseline_ (mV)	41.12 ± 2.87 ^a^	43.14 ± 3.37 ^a^	40.59 ± 3.94 ^a^
(Day 168)	CMAP_min_ (mV)	0.58 ± 0.12 ^a^	3.19 ± 1.60 ^b^	1.11 ± 0.57 ^a^
	CMAP_AAC_ (mV·day)	2752 ± 108 ^c^	2009 ± 298 ^a^	2377 ± 263 ^b^

CMAP_baseline_, action potential on the ipsilateral hind limb muscles prior to each repeated BoNT/A injection; CMAP_min_, lowest action potential recorded; CMAP_AAC_, cumulative action potential losses per injection cycle; Data represents mean ± standard deviation; One-way ANOVA with Tukey’s HSD test was used to compare values among BoNT/A formulations (mean values with different superscript letter are significantly different at α = 0.05).

**Table 3:** Summary of botulinum toxin type A formulations used in the study.

Product	Batch (Expiry Date)	BoNT/A Form	Dosage Per Vial ^a^	Preparation	Excipients ^b^
Botox^®^	C5394C3 (Sept. 2021)	900 kDa complex	100 U	Vacuum-dried	0.5 mg HSA 0.9 mg NaCl
Xeomin^®^	925559 (Sept. 2023)	150 kDa NAP-free	100 U	Lyophilized	1.0 mg HSA 4.7 mg sucrose
Coretox^®^	NSA20011 (May 2023)	150 kDa NAP-free	100 U	Lyophilized	L-methionine (q.s.) Polysorbate 20 (q.s.) 3.0 mg sucrose 0.9 mg NaCl

^a^ Dosage in units (U) based on the product information provided for each BoNT/A formulation. ^b^ Excipients comes in the same vial as the BoNT/A drug product and does not include excipients present in the reconstitution buffers that comes with their respective product.NAP, neurotoxin-associated protein; HSA, human serum albumin; NaCl, sodium chloride; q.s., quantum sufficit.

## Data Availability

The data presented in this study are available in this article.
